# Signaling strategies of silver nanoparticles in optical and electrochemical biosensors: considering their potential for the point-of-care

**DOI:** 10.1007/s00604-023-05666-6

**Published:** 2023-02-15

**Authors:** Franziska Beck, Michael Loessl, Antje J. Baeumner

**Affiliations:** Institute of Analytical Chemistry, Chemo- and Biosensors, University of Regensburg, Regensburg, 93040 Germany

**Keywords:** Silver nanoparticles, Optical biosensors, Electrochemical biosensors, Point-of-care, Clinical analysis, Localized surface plasmon resonance

## Abstract

Silver nanoparticles (AgNPs) have long been overshadowed by gold NPs’ success in sensor and point-of-care (POC) applications. However, their unique physical, (electro)chemical, and optical properties make them excellently suited for such use, as long as their inherent higher instability toward oxidation is controlled. Recent advances in this field provide novel strategies that demonstrate that the AgNPs’ inherent capabilities improve sensor performance and enable the specific detection of analytes at low concentrations. We provide an overview of these advances by focusing on the nanosized Ag (in the range of 1-100 nm) properties with emphasis on optical and electrochemical biosensors. Furthermore, we critically assess their potential for point-of-care sensors discussing advantages as well as limitations for each detection technique. We can conclude that, indeed, strategies using AgNP are ready for sensitive POC applications; however, research focusing on the simplification of assay procedures is direly needed for AgNPs to make the successful jump into actual applications.

**Graphical abstract:**

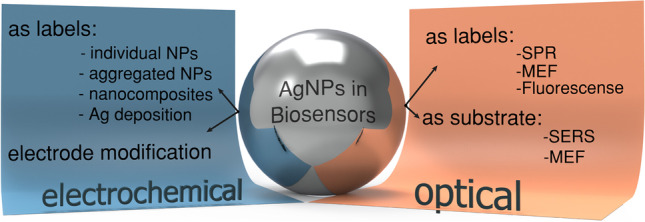

During the last 20 years, silver nanoparticles (AgNPs) gained increasing attention among the scientific community due to their unique physicochemical properties. However, biomedical properties of silver colloids were already exploited by the ancient Greeks and Romans, for example, in lotions or unguents [1]. Also, the antimicrobial effect of silver was used to protect vessels from bacterial growth and the food and drinks stored within from spoilage, long before the existence of microbes was found [2]. This effect is based on the interaction of silver ions with thiol type compounds in the vital enzymes and proteins of bacteria. With decreasing size of AgNPs, the surface contact area increases and with this the antimicrobial effect. In contrast to macro-sized silver, the nanoparticles are able to interact additionally with the cell membrane, which leads to its disruption and subsequent cell death [3]. Nowadays, with a production of 320 t per year AgNPs are the most abundant commercialized nano-compounds [4, 5]. They are widely used, for example, in cosmetics, textiles, medicinal products, or water decontamination [6]. In socks, for example, they inhibit the development of bad odors [7], while in bandages, they can improve soft tissue healing, preventing a bacterial infection [3]. More medical applications include anti-cancer therapy, dentistry [8], and the use as antidiabetic agent or vaccine adjuvant [9]. Only a minor part of the produced AgNPs is used in biosensors and serves as the signal generation and amplification system instead of enzymes, gold nanoparticles (AuNPs), and other nanocontainers. They are efficiently used in combination with optical and electrochemical transductions methods. Aside from their ubiquitous usage, toxicity concerns arose during the last years. It was found that silver nanoparticles play a major role in the generation of reactive oxygen species and oxidative stress in cells. Moreover, they are able to interact with different organs [4]. The toxic effect of AgNPs depends on various factors, e.g., size, shape, surface modification, dispersion, concentration, and cellular environment [10]. Also, the complex mode of action is not fully known and understood, yet. Therefore, the assessment of the grade of toxicity is a big challenge and has to be done very carefully.

Due to their general importance, AgNP synthesis (Fig. 1), surface modification, and characterization methods were extensively reviewed before. Therefore, it is shortly summarized in the following, but for further details, more specialized reviews are recommended, e.g., [9, 11–16].

**Fig. 1:**
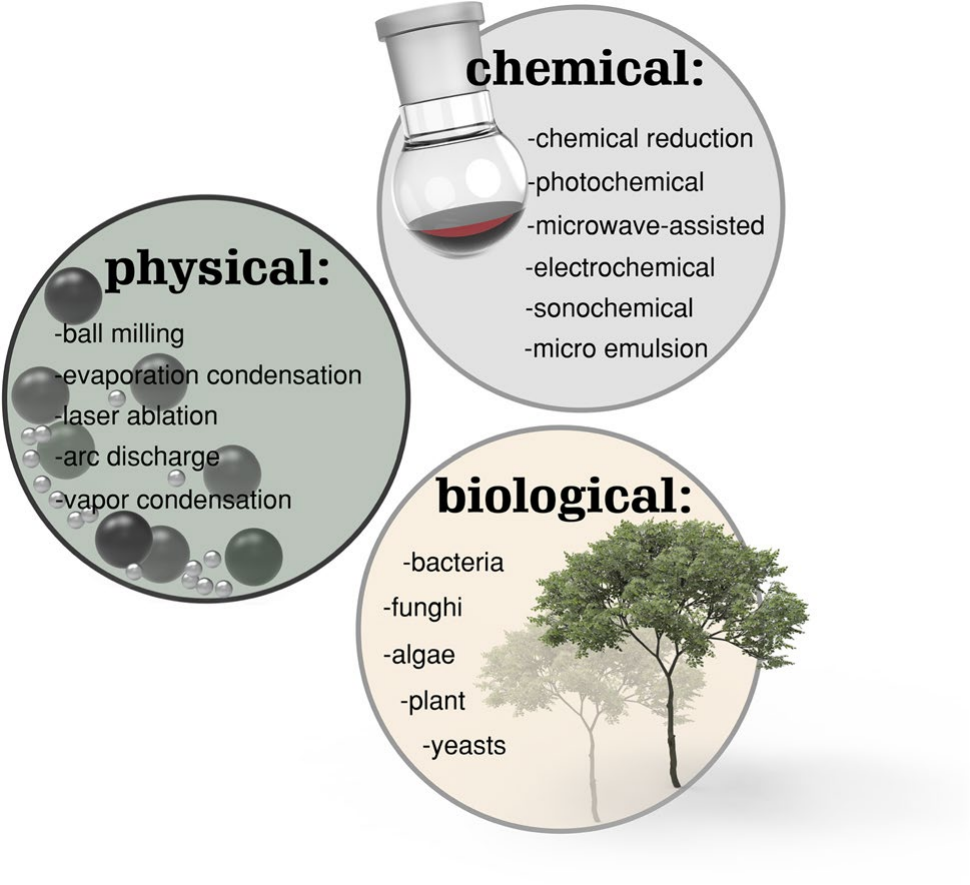
Summary of some possible synthesis methods for AgNPs with physical, chemical, or biological methods

AgNPs are generated either through “top-down” or “bottom-up” approaches. In the case of top-down strategies, also referred to as physical methods since no (bio)chemical reactions are used in their generation, the bulk material is broken down into fine nanoparticles, which are subsequently stabilized using colloidal protecting agents. The most common approaches are evaporation–condensation methods, in which the silver bulk material is evaporated and the generated supersaturated metal vapor condensates in the form of nanoparticles [17] and laser ablation of metal bulk material in organic or aqueous solvents [18]. Further physical top-down approaches include ball milling [19] or arc discharge [20, 21]. These methods are usually fast and easy to scale up, and the resulting nanoparticles are mostly free of hazardous chemicals. However, high energy consumption, solvent contamination, low yield, and especially a varying size distribution of the nanoparticles and their dispersion without aggregation can be challenging [22]. In the case of “bottom-up” approaches, the reduction of silver ions derived from precursor salts, e.g., AgNO_3_, forms nuclei via self-assembly followed by growth into nanoparticles. Methods to generate the energy needed for this reduction are photochemical [23], electrochemical [24], microwave-assisted [25], sonochemical [26], sol–gel processes [27], or reduction in a microemulsion. A frequently employed synthesis method is based on Brust and Schiffrin’s original work on the synthesis of gold nanoparticles [28]. The method involves a two-phase liquid–liquid phase system (water-toluene), and sodium borohydride is used as reducing agent [29]. Most of these chemical processes have the advantage of high yield, low cost, ease of production, and flexibility in nanoparticle (NP) shapes and sizes. However, the use of toxic chemicals for reduction leads to inadequate purity of the NPs and is untenable from an environmental point of view. Also, stabilization of the NPs against aggregation and control over their size are challenging and require the use of proper stabilizers. Green or “biological” synthesis of AgNP overcomes these disadvantages. Here, the natural plants, bacteria, fungi, algae, or yeast are used for the reduction of a precursor salt. These methods are environmentally friendly and pollution-free and avoid harsh conditions like high temperatures, pH, or strong reducing agents. Moreover, decreased time-demand, control over shape and size, simple scale-up and high stability, water-solubility, and density make these methods attractive in academia and industry [15, 16, 30, 31]. Post synthesis, AgNPs are characterized employing different methods, e.g., DLS, TEM, SEM, UV/Vis spectroscopy, EDX, FTIR, XRD, and XPS. A detailed insight into these methods is outside the scope of this review but can be found for example in [11].

In addition to fast, cost-efficient, and well controllable synthesis possibilities, metal nanoparticles offer a flexible, relatively inexpensive platform for signal amplification. They enable real-time detection of biomarkers in small sample volumes with a low limit of detection (LOD) and fairly simple procedures [32, 33], in comparison to enzyme-based or PCR-based signal amplification systems, and are therefore ideally suited for the point-of-care testing (POCT) [34]. Tests used by medical personnel can be observed increasingly on the market [35], and the Covid-19 pandemic has demonstrated that there is an enormous need also for POCT used by patients themselves. In addition to low LODs, other important features need to be addressed including low sample volume demand (especially for blood testing), no additional pipetting, fast response time, low cost and small size of the detection device, and storage of all chemicals in dry format on chip. Based on the high surface-to-volume ratio of AgNPs, they exhibit special optical and electrochemical properties, which render them ideal for the use in biosensors in combination with different transduction methods [11]. This review will focus on the progress of the use of silver nanoparticles in optical and electrochemical biosensors for clinical diagnostic in the last 4 years, especially assessing critically their potential for the use at the point-of-care.

## Silver nanoparticles in optical biosensors

**Fig. 2:**
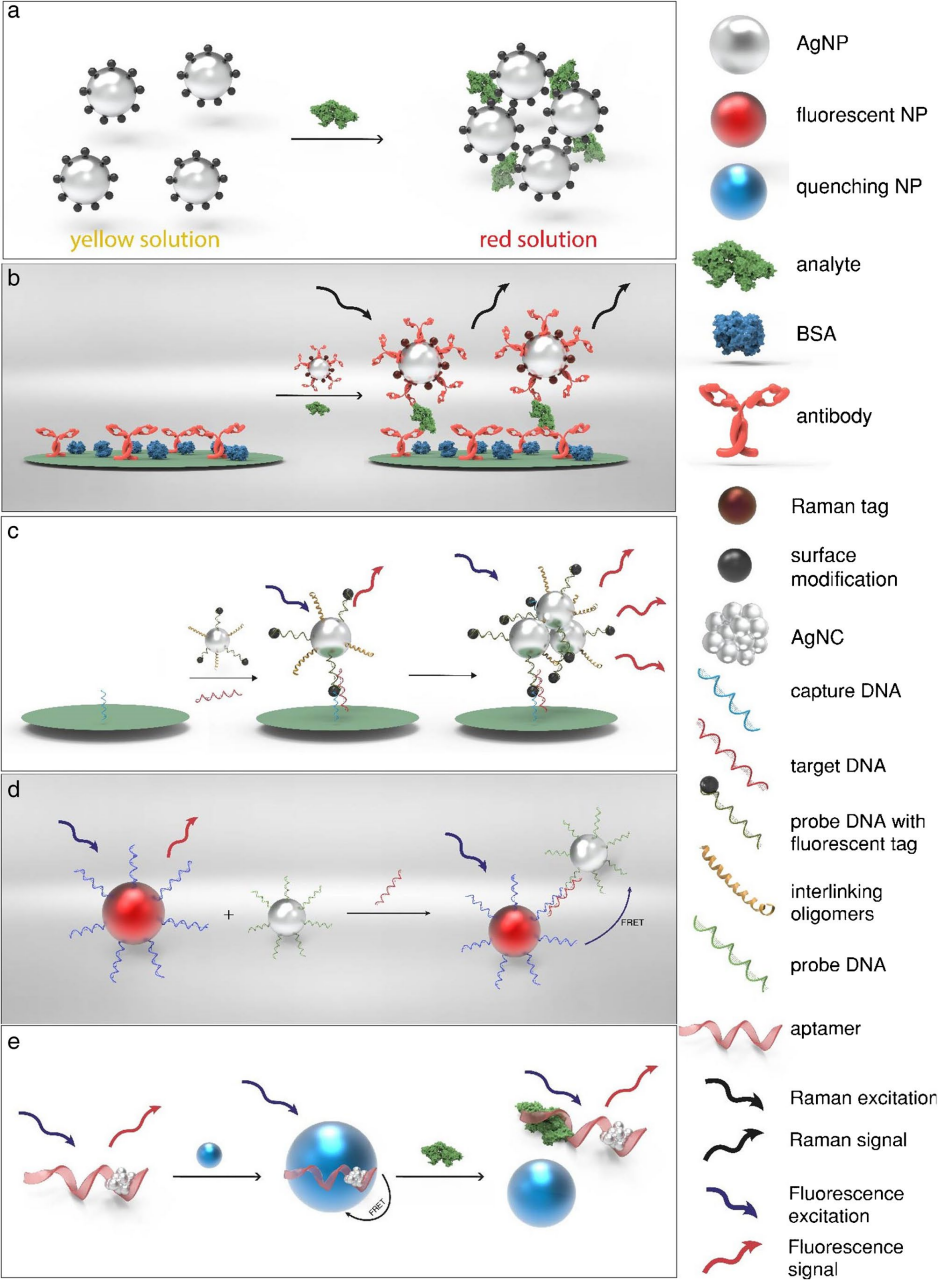
Schematic representation of the working principle of various optical biosensors using AgNPs based on **a** SPR, **b** SERS, **c** MEF, **d** FRET, or **e** the inherent fluorescent properties of AgNCs

AgNPs exhibit various special optical properties that can be harnessed for detection by transducers commonly used in biosensors (Fig. 2). (i) Their freely moving electrons when excited by visible light cause surface plasmon resonance (SPR) [36] which in turn leads to a strong absorption which depends on shape, size, and dispersion of the AgNPs [14]. Therefore, biosensors detect either the plasmon band shift upon aggregation or changes in the local dielectric constant/refractive index (RI) due to mere binding to the analyte. (ii) Their inherent ability to enhance the Raman-scattering of molecules in spatial proximity of their surface can be employed resulting in SERS-based sensors [37]. (iii) AgNPs provoke metal-enhanced fluorescence (MEF) when in a short distance (5–90 nm) to a fluorophore. As they increase the rate of excitation and emission, and enable additional electronic configurations of the fluorophore, quantum yield, photostability, and overall sensitivity of the fluorescence sensor are increased [38]. (iv) With a high molar extinction coefficient and absorbance in the visible range, AgNPs are considered optimal fluorescence quencher [39]. This enables assay strategies based on Foerster resonance energy transfer (FRET). (v) Also, the intrinsic fluorescence of Ag nanoclusters (AgNCs) can be used for transduction.

### SPR-based biosensors

Surface plasmon resonance (SPR) is the collective oscillation of conductive electrons, which origins from the acceleration of the electrons by the incident light in combination with restoring forces due to the polarization of particle and environment and confinement of electrons to dimensions smaller than the wavelength of light [40]. The dependence of the SPR band on size, shape, interparticle space, surface charge, and nature of the surrounding medium [2] can be exploited as transduction principle. Biosensors based on Ag nanoarchitectured surfaces (e.g., nanospheres, nanoholes, nanoarrays) measure analyte-dependent RI changes [37] whereas those using AgNPs based on the SPR-band change upon analyte-induced de-/aggregation of the silver nanoparticles (Fig. 2, a). Thus, all molecules that induce aggregation via interaction of functional groups with the AgNP surface ligand or that change the dielectric environment of the AgNP can be target analytes [41].

Here, silver is superior to other NPs due to its high molar extinction coefficient and narrow SPR-band in the visible region [2]. With decreasing interparticle distance, the plasmonic fields of the individual particles overlap, and the absorption shifts to a higher wavelength or decreases. This principle has been employed for years for the detection of various analytes and was reviewed extensively before [2]. It lends itself very well for clinical diagnostics and the POC due to the simple, visual, or colorimetric read-out. It has recently been adopted toward the specific detection of biothiols [42, 43], DNA [44], or small molecules like adenosine [45] where quantification is obtainable through a change of absorption or even absorption ratio on two different wavelengths.

Wang et al.[46]. also introduced the possibility of in situ generation of AgNPs as SPR-label. In the presence of the target miRNA, single strands of DNA could unfold and form dsDNA via hybridization chain reactions. Silver ions are then inserted into the DNA double strand, and, upon introduction of a reducing agent, silver nanoparticles are formed which in turn led to a change in SPR angle. Benefits of this technique include not time-consuming and costly modification of the nanoparticle with biomolecules.

The major drawbacks of SPR-based sensors include the typically large reagent volume needed for this format, e.g., 150 µL [44] in microtiter plate (MTP) assays and up to 5 mL [42] in cuvettes. Aside from cost and environmental drawbacks related to large volumes, such formats also often require large sample volumes to promote effective aggregation. MTP assays require 50 µL of sample making it impossible to handle finger prick samples, which usually express blood volumes ≤ 10 µL, without further dilution [47]. Other biofluids are better suited for these kinds of aggregation assays, such as urine or saliva, because they are readily available in big volumes. Recently, Shariati and Khayatian transferred these aggregation assays into a small paper-based device [48], negating sample volume challenges. This paves the way towards a new generation of AgNP aggregation assays, making them applicable even to finger prick samples. Secondly, finely tuned surface modification adapted to not only the analyte, but also the desirable matrix is typically challenging. In fact, aggregation of not well-adjusted AgNPs is mostly influenced by electrolyte concentration or pH, while the presence of biomolecules can inhibit aggregation altogether [49]. Finally, detection in turbid samples is not possible, which is a common problem with absorbance measurements, and showcases a common need for careful sample preparation strategies.

While using a photometer for the read-out might be easy in a lab, it adds additional cost, size, and complexity to such a self-testing approach. Smartphone read-out can be a solution, but was known to be problematic due to variations in lighting, in handling by the patient or in the smartphones themselves. However, recent advances in smartphone read-out by Choi et al. [50] demonstrated that with sophisticated mathematical corrections such as RGB analysis or internal standards these problems can be overcome. For patient handling, a combination of smartphone read-out with paper microfluidics is advantageous for the optical aggregation assay and suggesting that AgNP aggregation assays will be highly useful at the POC in the future.

### Biosensors based on surface-enhanced Raman scattering (SERS)

Surface-enhanced Raman scattering (SERS) is the chemical and electromagnetic enhancement of Raman signal of a molecule in close proximity or adsorbed to a SERS substrate. It has been identified as a unique signal generation strategy in biosensors as it offers high anti-interference stability due to highly specific fingerprint regions with narrow peaks [51], high multiplexing capabilities, rapidity, low sample volume requirements, nondestructivity, and low background noise [52, 53]. AgNPs and combinations thereof with other materials such as with silicon chips or carbon materials are an ideal SERS-active substrate due to their high optical reflectivity onto which a sample is deposited (referred to as intrinsic SERS). Their composition, size, shape, and surface roughness influence the SPR band and with it its SERS efficiency [37]. This was employed for the detection of glutathione [54], miRNA [53], ATP, and bacteria [55]. These approaches enable easy implementation, label-free procedures, and little sample preparation [56], but suffer from slow kinetics due to diffusion limitations [51] and hence overall slow sensor responses. Furthermore, data analysis of the directly measured sample is usually complex and requires advanced statistical methods such as principal component analysis (PCA) or other machine learning techniques, so that much software engineering is needed to make it applicable for use with health care professionals [56]. Also the preparation of a suitable SERS substrate is challenging as not well-defined structures can account for insufficient enhancement factors and poor stability of the SERS signal, while complicated fabrication procedures and expensive chemicals lead to higher time and money consumption and lower reproducibility [54, 57]. Here, recent research by Bu et al. [54] demonstrated advances in nanoengineering and synthesis procedures that provide a solution to these challenges by simplifying production of SERS substrates.

Alternatively, researchers employed modified nanoparticles (SERS-tags) as labels [56] in extrinsic SERS sensing (Fig. 2, b). For example, AgNPs with Raman reporter probe on their surface were recently used for the detection of proteins [51], micro RNA (miRNA) [52], and DNA [58]. These publications demonstrate highly sensitive, reliable, and simple quantification of biomolecules utilizing SERS as transduction principle. Quarin et al. summarized various recent SERS sensing principles, such as aggregation-based, paper-based, magnetic-capture, and homogeneous assays, with their respective special advantages and disadvantages for POCT in their review [56]. In general, the extrinsic SERS sensing demonstrates a higher potential to be used for POC, as it is simply implemented in already established assays as an alternative, superior detection method [56].

Recently, the effect of photoinduced-enhanced Raman spectroscopy (PIERS) gained attention due to its potential to greatly increase sensitivity beyond the SERS effect [59]. PIERS is based on the photoexcitation of a semiconductor material, namely, TiO_2_, which enhances Raman scattering at the site of silver or gold nanoparticles [59]. The photoexcited material can charge the respective nanoparticle and, in presence of a sample, charge-transfer between the sample and the NP lead to the PIERS effect. This effect has already been exploited in first biosensors to sensitively detect ATP, thrombin, or cocaine [60]. As the PIERS effect was found rather recently, implementation in biosensors is still scarce; however, considering the great enhancement in sensitivity compared to the normal SERS effect, this effect has great potential for future sensing applications.

In general, SERS analyses often suffer from poor repeatability and stability of the signal, which can cause inaccurate quantification of the analyte [55, 57]. This makes quantitative SERS difficult, calling for internal standards, a highly ordered substrate, and/or shielding from external influences [55], as demonstrated through ratiometric sensing [53]. Interestingly, Chen et al. also substituted the Raman microscope with a portable spectrometer, acknowledging that it in combination with multistep detection leads to worse sensitivity and longer times. Unfortunately, this common, truly important challenge that hinders SERS advance into the POC is seldomly picked up in new publications. Instead, most focus on developing new, ultrasensitive SERS substrates or tags as proof of principle rather than simplifying the method towards the use at the POC. Therefore, while the majority of the publications shown here were already applied for clinical samples, none go the next step, i.e., validation of the method and integration into end user devices [56].

### Metal-enhanced fluorescence-based biosensors

Fluorescence is a commonly used transduction technique in biosensors due to its versatility, simplicity, sensitivity, and multiplexing capability [38]. Aside from the high demands of a fluorescence detector, autofluorescence of the samples, low quantum yield, and photobleaching of traditional fluorescent labels hinder ultra-sensitive fluorescent detection in a POC application [2]. Enhancing the fluorescence via close proximity (5–90 nm) of the fluorescent dye to a colloidal metal surface, which increases the rate of excitation and emission and enables additional electronic configurations of the fluorophore, is therefore one of the research strategies pursued. It increases quantum yield and photostability of the fluorophore and after all sensitivity of the sensor. The exact metal-enhanced fluorescence (MEF) mechanism based on localized surface plasmon resonance was discussed previously [38]. AgNPs are frequently employed for this purpose and indeed offer better performance than AuNPs due to their strong SPR, which spans over a broad region of the visible spectrum [61]. Analogous to the SERS sensors, silver nanoparticles can be used as 2D substrate, for example, in combination with a quartz matrix [62]. However, if no sophisticated patterning techniques are used, the random distribution of nanoparticles and nanostructures often hinders the reproducibility of such sensors. Further limiting factors are analogous to the 2D SERS substrates and include slow diffusion to a rigid surface [38] and the comparatively low surface area [63], which hinders the immobilization of large amounts of biorecognition elements and with that lowers the performance. Researchers try to solve these problems, for example, by using AgNPs in combination with electrospun nanofibers as 3D substrate [63]. On the other hand, AgNPs can also be used as colloidal label with an inert organic or inorganic shell [61, 64]. This core–shell structure ensures the exact distance between metal surface and fluorescent dye and simultaneously improves stability, biocompatibility, and dispersability [38]. Aggregation assays are often used (Fig. 2, c), such as for the detection of DNA [61], polysaccharides, or enzymes, like heparin and heparinase [64]. They afford ultrasensitive and low-background fluorescent detection [38]; however, absorption and light scattering of other nanoparticles in the dispersion reduces the effectivity of the colloidal MEF in comparison to 2D approaches [38].

Manufacturing of MEF tags and substrates is rather complicated as an exact distance between metal, and fluorophore is crucial. If the parties are too close together, the AgNPs quench the emission, while the effect does not appear for too long distances. At first glance, this hinders AgNP-induced MEF application to the POC, yet, with well-designed systems providing signal amplification of a factor of multiple hundreds [38], these efforts should be worthwhile. This leads to the common final challenge, i.e., matrix effects on the MEF signal. Typically, extensive sample preparation is required which cannot be implemented in the POC. Li et al. [64] suggested dilution of serum as a solution to this challenge and did their analyses in 1% human serum. However, Kim et al. [65] successfully used MEF-enhanced detection in a microfluidic device. As the implementation in such miniaturized devices is an important step in the development of POC sensors, it can be assumed that the first fully functioning MEF POC devices emerge in the near future.

### Other fluorescent sensors

Ag nanospheres with their broad SPR band, high absorption coefficients, and anisotropic shape are ideal fluorescence quenchers [39]. Due to their shape, they do not have a defined dipole moment, which enables energy transfer in any orientation, and the efficiency of FRET increases [39]. This “superquenching,” exhibiting Stern–Volmer quenching constants several orders of magnitude larger than those of normal quenching processes, has been exploited to develop sensitive, rapid, and homogeneous biosensors [39]. In the last years, AgNPs were employed as quencher in combination with various other fluorescent nanomaterials like metal–organic frameworks [66], carbon dots [67], or silica nanoparticles [68]. These FRET sensors can either be employed for analytes which are able to cross-link donor and acceptor (Fig. 2, d), such as miRNA [66], or analytes which are able to reduce Ag^+^ and form the quenching AgNPs in situ, e.g., ascorbic acid [67] or dopamine [68]. These sensors can be developed in “turn-on” or “turn-off” mode. However, the AgNPs as quenchers are rarely employed for clinical analytes and rather for environmental mercury monitoring. In fact, most published work is still in the proof-of-principle stage, trying to fully understand mechanisms and influencing factors, rather than adapting specific assays to the POC [39]. Yet, this method has true potential for future POC developments due to its simplicity, sensitivity, rapidity [67], and the various combination possibilities of materials and methods and is only limited by the properties of the fluorescent dye such as photostability, quantum yield, and photobleaching.

Recently, silver nanoclusters (AgNCs), made of several to tens of atoms resulting in a size in the range of the Fermi wavelength of electrons, have been shown to overcome many of these limitations [69]. They possess an emission varying between blue and the near-IR depending on the size of the cluster, which makes them ideal for multiplexing [37]. They have a high quantum yield, narrow photoluminescence bands, and excellent biocompatibility [70]. Also, their simple synthesis protocol is usually performed by reduction of a silver salt in presence of a DNA template, and their emission can be tuned by changing the sequence and length of the oligonucleotide [71]. Most recent sensing examples include RNA or miRNA detection [69, 72], ELISAs [71], or dopamine [73] or even combine the DNA-templated synthesis with aptamers (Fig. 2, e) [70]. The applicability of AgNCs in biological matrices was only tested by Jiang et al. [69], while the other publications can be seen more as a proof of principle. Thus, the overall stability of AgNCs in biological fluids still has to be evaluated thoroughly. Moreover, many assays rely on the in situ synthesis of the AgNCs, which is not possible considering a POC, self-testing approach due to multistep addition of chemicals. However, if AgNC long-term stability can be confirmed, they will have a great potential of replacing traditional, organic fluorescent markers.

## Silver nanoparticles in electrochemical biosensors

Electrochemical biosensors tend to be preferred over optical ones in current POC research, since they show high sensitivity, cost-effectiveness, simplicity, anti-interference ability, and the potential for real-time analysis, which is particularly interesting for clinical analysis of biological fluids [74–76]. Simultaneously, they have a higher potential for miniaturization, because no optical components or minimum path lengths are needed [77, 78]. Therefore, sensors with electrochemical detection are frequently used in food quality monitoring, biomedical research, clinical diagnostic, and environmental monitoring [33]. Although less investigated in the electrochemical field than for optical sensing, AgNPs are of special interest as they have the highest conductivity of all metals; high electrochemical activity, i.e., low oxidation potential and high electron transfer rates; and catalytic activity towards certain analytes [79, 80].

### Silver nanoparticles as labels

The characteristic Ag/AgCl solid-state reaction of AgNPs is used for their quantification as labels. Oxidation of Ag is directly monitored via voltammetric methods, like linear sweep voltammetry (LSV), square wave voltammetry, or differential pulse voltammetry. AgNPs are also dissolved for sensitive detection via anodic stripping voltammetry (ASV) [79]. Due to their high electrochemical activity, the oxidation and reduction are possible using a low potential and without the use of additional hazardous chemicals. This minimizes the potential interferences and ensures a reaction outside of the potential region, where the electrochemical reaction of dissolved oxygen occurs [81], much in contrast to the 1.25 V needed in the presence of HCl [82] for the oxidative dissolution of AuNPs. Moreover, their relatively sharp peaks enable precise and sensitive detection [83]. Thus, AgNPs are highly advantageous as label in electrochemical sensors in comparison to, for example, gold nanoparticles or quantum dots and are used employing four overall strategies.

#### Individual AgNPs

**Fig. 3:**
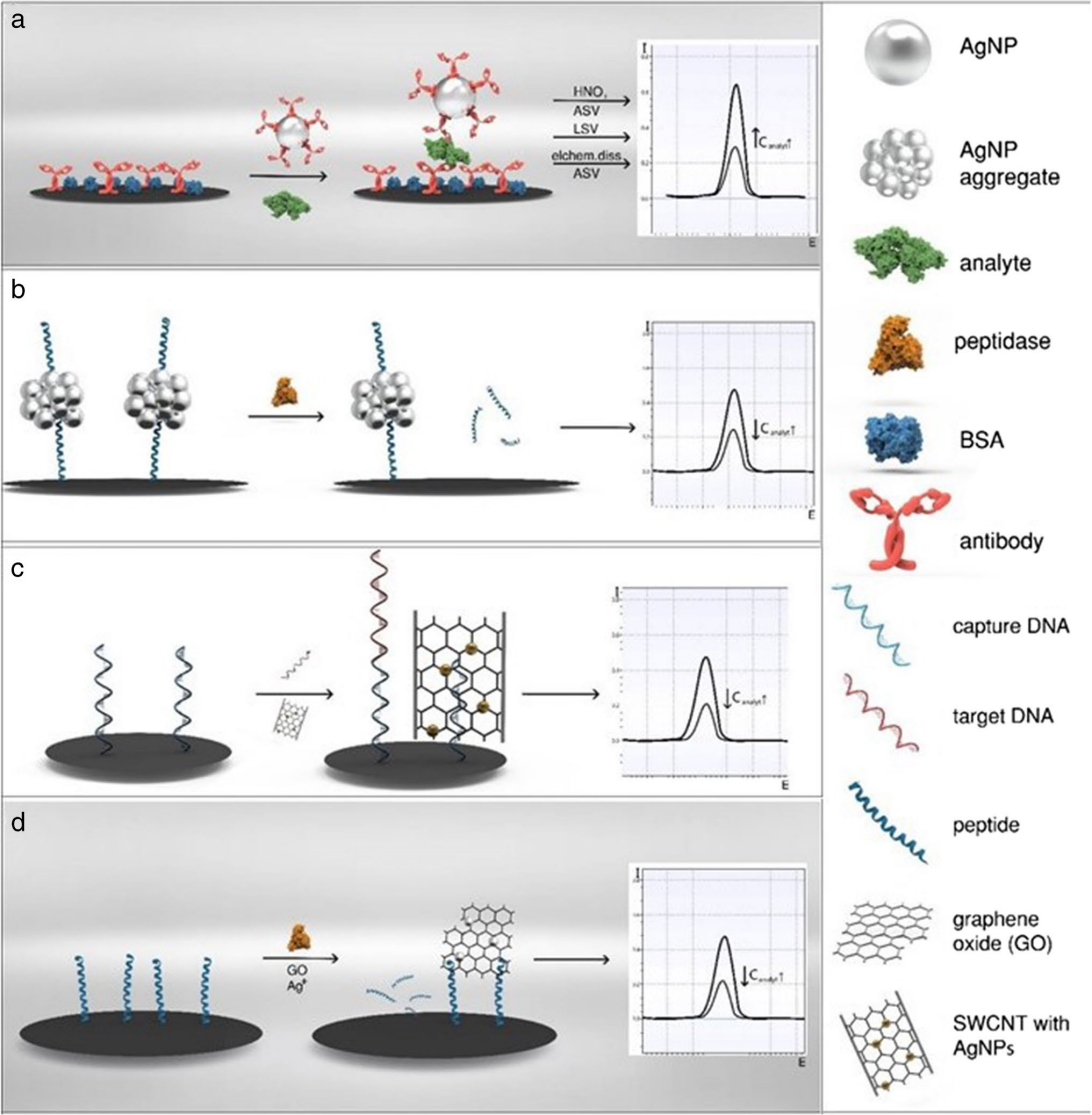
Schematic representation of a bioassay using **a** individual AgNPs with chemical dissolution, direct LSV, and electrochemical dissolution, **b** AgNP aggregates or **c** nanocomposites as electrochemical label, or **d** in situ synthesis of AgNPs on graphene oxide

Since one individual AgNP consists of thousands of silver atoms, considerable signal-amplification compared to the use of single electrochemical labels is achieved (Fig. 3, a). Traditionally, AgNPs were dissolved using strong acids like HNO_3_, preconcentrated on the transducer by reduction and then oxidatively stripped off the surface [74, 78]. This method is very sensitive, since the chemical treatment dissolves the whole particle, and can potentially clean the electrode, and anodic stripping voltammetry is known to be used for trace analysis. Still, as the chemical dissolution uses harsh chemicals, is time-consuming, and can be the origin of mistakes, its application especially in the POC is rather problematic [84]. To overcome these limitations, a number of researchers monitor directly the oxidation of the Ag in KCl electrolyte via linear sweep voltammetry, applying it to clinical analytes, e.g., prostate-specific antigen (PSA) [77], enzyme activity [76], or DNA [85]. Further improvements employ various electrochemical dissolution strategies [86, 87] resulting in a combined preconcentration and LSV stripping analysis offering fast and simple procedures, one-step assays, and even chip-based approaches [88]. Thus, this technology is ready for the POC. Yet, challenges occur when the AgNPs need to be modified with biorecognition elements or stabilizing shells as these may hinder the electrochemical conversion for electrochemical dissolution or direct detection through the formation of a electrically insulating layer around the particle. This increases the distance between electrode and AgNP and with it the necessary potential until no conversion is possible at all [89].

AgNP modification can easily be achieved via physical adsorption based on the interactions between the silver atoms and thiol or amine residues of proteins. Problems tend to occur during long-term storage, where additives such as BSA or other biologicals are competing for the AgNPs and can replace the biorecognition molecules [87]. Pollok et al. [87] solved this issue by employing a heterobifunctional cross-linker. Other important aspects related to long-term storage are the prevention of Ag oxidation by air oxygen and the colloidal stability of the labels in a biological environment. Strategies have been suggested most recently such as drying in stabilizing matrices [90] or stabilizing shells around the AgNP [84]. Without paying attention to these long-term effects of AgNP as electrochemical label, no application to POC is possible.

#### AgNP aggregates and nanocomposites

A simple approach used to increase AgNP signaling efficiency is suggested through the creation of AgNP aggregates (Fig. 3, b). Aggregates were specifically manufactured, for example, by reaction with CB[8] [91], DNA [92], or peptide [93] templated synthesis. However, analogous to the SPR aggregation sensor, finely tuned surfaces and methods are of immense importance as the signals vary greatly upon unspecific or uncontrolled aggregation, which typically occur in biological matrices. Considering a POC application, this makes any one-step procedure impossible [49]. Nonetheless, the advantages with respect to limits of detection obtainable are obvious, e.g., comparing AgNP [77] with AgNP aggregates [92] for PSA detection (0.11 pg·mL^−1^ vs. 33 fg·mL^−1^), and warrant further research efforts toward their controlled use. A somewhat more complex approach is suggested through the generation of nanocomposites (Fig. 3, c), where AgNPs are combined with other nanomaterials such as nanofibers [94], carbon nanotubes [95], graphene oxide [96], C_60_-AuNPs [97], or cubes [81]. While in some instances the second nanomaterial may only be a physical support, special attention deserve carbon nanocomposites as these are easy to synthesize due to the natural affinity of Ag^+^ ions to carboxyl and carbonyl groups [95], serving as effective nucleation site for AgNP synthesis. The advantages of both materials can thus be combined, and various analytes can be detected ranging from whole cells [94] to small molecules like miRNA [95]. For POC applications, there are no specific limitations to the use of nanocomposites as long as the synthesis and assay can be performed in a fast and cost-effective way and the nanocomposites exhibit sufficient stability. Furthermore, nanocomposites consisting of more than two materials are emerging, to finetune biocompatibility, reactivity, and stability of the material. Wang et al. [98] developed a nanocomposite consisting of reduced graphene oxide (rGO), polydopamine (PDA), AgNPs, and Ti^4+^-cations. The rGO-PDA composite ensured environmental stability and biocompatibility, and AgNPs are used to generate the voltammetric response, while the Ti^4+^-cations are designed for specifically recognize phosphopeptides as a marker for protein kinease activity. Due to the large flexibility of nanocomposites, which are able to overcome some of the challenges when working with AgNPs, they are a promising approach for a future generation of sensitive biosensors.

#### Promoted reduction of Ag+ and silver deposition

The aforementioned carbonaceous nanocomposite can also be synthesized in situ during the assay (Fig. 3 d). In addition, other strategies exist for the in situ synthesis of AgNPs such as hydrazine-modified AuNPs [99], DNA [100, 101], or alkaline phosphatase [102]. While the reaction times can be shortened considerably by choice of a proper catalyzing system (e.g., with C_60_-AuNPs to only 11 min [97]), most often long reaction times of 1 h are needed for the AgNP synthesis as the last step of the assay [96, 101]. Together with the need of a further addition of solutions in the experimental procedure, these methods can only be considered difficult for a POC self-testing procedure, and the publications shown so far do not convince with lower limits of detection or sensitivities in comparison to the standard AgNP nanocomposites.

### Silver nanoparticles for the modification of the electrode material

**Fig. 4:**
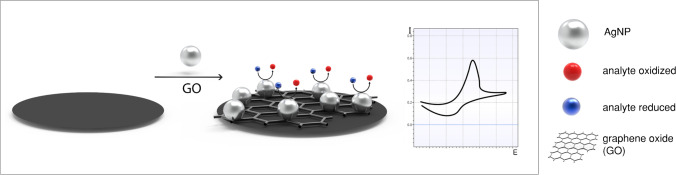
Schematic representation of an electrode modification designed to facilitate the oxidation of an analyte molecule

AgNPs are used just like other metal NPs to increase the active electrode area and also to provide additional reactivity to simplify or enhance the oxidation or reduction of an analyte (Fig. 4). The advantages are numerous and similar to those found for other metal NPs, and it is not always clear whether silver is the optimal material indeed or can be replaced by other metal NPs. For example, a glassy-carbon electrode was modified using eco-friendly synthesized mucilage-AgNPs to enable the detection of glucose in human blood samples [103]. By covering the electrode with AgNPs, the mass transport to the surface increases due to convergent rather than linear diffusion [104]. Moreover, more exposed crystal planes in comparison to the bulk material can lead to current improvement, and covering a low-cost electrode, like screen-printed carbon electrode, with only a fraction of the expensive metal is highly cost efficient in comparison to using a silver electrode [104]. These effects are also exploited in inkjet-printed electrodes. AgNP-based ink is already commercially available and can thereby easily be used to form electrodes [105]. Such electrodes can, for example, be used to monitor bacteriophage contaminations in food samples [105]. Silver also possesses electrocatalytic properties which can be exploited for the direct detection of hydrogen peroxide [106] or ascorbic acid [75] on AgNP-modified electrodes. The sensitivity of these sensors benefited simultaneously from better electron transfer and larger active surface area.

Similarly, AgNP-coated electrodes were found to interact with the redox active center of enzymes enabling direct electron transfer. Moreover, they form a microenvironment that is similar to the native one for redox enzymes, which prevents denaturation of the proteins on the plain electrode surface and thus, facilitate enzyme immobilization [80]. The modification of electrodes with AgNPs is usually done via electrodeposition, which is low-cost, highly productive, and readily adaptable to mass-production [80]. However, the building of such composite electrodes increases the complexity of redox reactions happening on the surface which gives rise to measuring artifacts and selectivity and specificity issues [73]. To avoid false results due to measuring artifacts, a fundamental theoretical knowledge of the processes on the electrode is needed, as already Campbell and Compton [104] discussed in their review 2010. Meanwhile, researchers, such as Khalifa et al. [103], focus more on a fundamental understanding of the processes rather than merely empirical studies. If specificity and stability of the sensor can be guaranteed, for example, by careful surface modification, and the detection works also in biological media, this method would indeed be good for a POC self-testing approach due to the ease of electrode production and handling, high-cost efficiency, and sensitivity.

## Challenges in the use of AgNPs

### Addressing stability issues of AgNPs

AgNPs were disregarded as appropriate material for biosensors for a long time due to stability and toxicity concerns. Effective ways to enhance the colloidal stability of AgNPs have been known for many years, although the surface modification has to be adjusted to the respective detection technique and biological matrix [84, 107]. In fact, in the case of biosensor applications, with AgNPs typically modified with biomolecules, prevention of aggregation is unproblematic. Nonetheless, the stabilization of the AgNP colloid against aggregation is reached by electrostatic (e.g., H_2_, citrate), steric (e.g. PVP), or electrosteric (e.g., BPEI) surface modifiers. Sterically and electrosterically stabilized particles demonstrated highest resistance to aggregation even at high ionic strengths, electrostatically stabilized AgNP aggregation kinetics followed the DLVO theory [107]. A more dominant issue results from the highly active nature of Ag as it is easily oxidized by air oxygen, which changes the amount of Ag through storage and is thus highly unfavorable for any POC application. This occurs in liquid and in dried formulations, which makes it difficult to implement in self-testing tests. Two strategies have been employed recently for the protection from oxidation: drying in a protective matrix with oxygen scavenger [90] or surface modifications [14] of the AgNPs themselves. In the case of the latter, AgNP properties are altered depending on coating material and layer thickness [14] and therefore must be very carefully designed. For example, inert polymer coatings can stabilize optically used AgNPs, while the shell potentially hinders electrochemical conversion. In the case of protective drying matrices, these can be useful in electrochemical transduction methods but have a possibly negative effect on optical detection strategies, for example, via hindrance of aggregation (for SPR-based techniques) or increased turbidity and scattering (for the optical detection itself). Thus, not surprising, no universal solution exists, and stabilization processes of the AgNPs must not only be carefully chosen with respect to the application but also early on in the development of the POC system. Some publications start addressing this issue, but more attention needs to be put forward to find long-term and smart solutions.

### Toxicity of silver nanoparticles

Silver nanoparticles employ well-documented antimicrobial and antiviral properties. While these properties are exploited in a multitude of different applications, ranging from odor-inhibiting socks to antiviral face masks, they also raise concern for their application in biosensors. Concerns are raised regarding the toxic effect AgNPs and dissolved Ag^+^ ions can have on biomolecules [108] which would result in a destruction of the important, adjacent biorecognition element. Moreover, while in most POC devices no direct contact between the respective test subject and AgNPs should be occurring, nontoxic materials are obviously favored to reduce the risk of accidents and potential environmental harm upon discarding of the test systems. Overall, the toxicity of silver nanoparticles is not fully understood, and ongoing research is carried out studying AgNPs of varying shapes, sizes, and surface modifications. Based on such studies, it is known that high concentrations of Ag ions lead to decreased cell viability [109–111] which suggests that more stable nanoparticles are less toxic due to decreased in situ oxidation of the particles. However, Pakrashi et al. showed that bioaccumulated nanoparticles had a weaker, but longer negative effect on living organisms compared to silver ions [111]. Furthermore, it is found that AgNPs of different sizes and with different surface modifications have different toxicity mechanisms [112]. Similar to AgNP, the toxicity of nanocomposites including silver also varies depending on the exact composition, shape, and size. Titov et al. found a low toxicity in silver-selenide nanoparticles encapsulated in a polymer matrix [113]. On the other hand, however, the frequently used nanocomposite consisting of graphene oxide and AgNPs was reportedly even more toxic compared to its pristine counterparts [114]. Therefore, the toxicity of specific AgNPs has to be monitored carefully prior to their implementation into commercial POC tests. For further information on toxicity, more detailed information can be found in several reviews, such as [115–118].

## Future perspective for AgNPs in biosensors

As evident from the previous chapters, the challenge to fully incorporate AgNPs in biosensors remains which needs to be addressed (also summarized in Table 1), foremost of which are stability and toxicity of the nanoparticles before they would be routinely incorporated into POC sensors. Yet, it has also been shown that not all AgNPs behave the same and result in rather varying degree of toxicity, for example. Thus, considering the multitude of well-established synthesis techniques and a variety of surface modifications, it would appear as if these properties are controllable and tailorable so that their employment in commercial diagnostic tests can be appropriate already considering their in-part superior analytical performance.

**Table 1:** Overview over advantages, limitations and possible solutions for the optical and electrochemical transduction techniques employing AgNPs with corresponding literature examples

Transduction technique		Advantages	Limitations	Possible solutions	Examples
Optical	SPR	• Wide number of possible target analytes • Simple visual/colorimetric read-out	• High volume demand • Unspecific aggregation • Strongly colored/turbid samples	• Paper-based devices • Finely tuned surfaces • Smartphone-assisted readout	[42, 44, 45]
	SERS	• Specific fingerprint region • Multiplexing capabilities • Rapid, nondestructive • Low volume demand and background • Easy implementation • Label-free, little sample preparation (intrinsic) • Sensitive, reliable, simple (extrinsic)	• Low repeatability • Sensitivity and rapidity suffer from portable Raman spectrometer • Data analysis, slow diffusion, challenging preparation (intrinsic)	• Internal standards • Shielding from environment • Advances in nanoengineering	intrinsic: [53–55] extrinsic: [51, 52, 58]
	MEF	• High versatility, simplicity, multiplexing capability • Increased quantum yield, photostability, sensitivity • Ultrasensitive, low-background (colloidal)	• Low reproducibility vs time-consuming, costly fabrication (2D) • Slow diffusion, low surface area hinders immobilization of large amounts of biomolecules (2D) • Inner filter effect (colloidal) • Complicated manufacturing process for substrates and tags • Rather low fluorescent amplification • Measurement in biological fluids challenging • Rather expensive instrumentation	• Use of 3D substrates, *e.g.* nanofibers • Advances in nanoengineering • Finely tuned surfaces • Well-thought-through systems	[61, 63, 64]
	FRET	• Extremely effective quenching (“superquenching”) • Simplicity, rapidity, sensitivity • Various possible combinations of materials	• Limits of employed fluorescent dye, *e.g.* photostability, quantum yield, photobleaching • Research still in proof-of-principle stage	• Adaption of principles to the POC	[66–68]
	Fluorescence of AgNC	• Tunable emission, high multiplexing capabilities • Simple synthesis • High quantum yield, narrow emission bands, excellent biocompatibility	• Unknown stability in biological fluids • In situ synthesis of AgNCs	• More application examples in biological matrices • Confirmation of long-term stability of AgNCs	[69, 71, 72]
Electrochemical	Ag NPs, aggregates, or nanocomposites as labels	• Simple, sensitive, fast, low-cost, high throughput, easy miniaturization • Possible one-step assay • Huge signal amplification • Flexibility due to big amount of possible combinations (nanocomposites)	• Surface modification may hinder electrochemical conversion • Modification via physisorption decreases performance, stability • Prevention of Ag oxidation, (unspecific) AgNP aggregation	• Use of specific linkers • Drying in stabilizing matrices, stabilizing shells	[90, 92, 95]
	Ag^+^ reduction, Ag deposition	• Combination with established enzyme systems	• Reagent addition after sample addition • Often long reaction times	• Proper catalyzing systems • Flow-injection analysis might enable automatic reagent addition	[96, 97, 99]
	AgNPs as electrode modifier	• Easy, highly cost-efficient electrode preparation, adaptable to mass-production • Ease of handling • High sensitivity • Stabilization of immobilized enzymes	• Increased complexity of reactions leads to measuring artifacts • Potentially difficult in biological medium • Selectivity and specificity issues	• Careful surface modification • More fundamental research for a better understanding of processes	[75, 103, 106]

To date, AuNPs, while overall occupying a smaller market share compared to AgNPs, are more frequently employed in commercially available biosensors [119]. Consequently, with a larger database and more available research experience, AuNPs keep being favored also in new research development. However, changes are on the horizon since AgNPs exhibit several properties preferable to their gold or other noble metal counterparts, such as the highest conductivity of all metals and high electrochemical activity, i.e., low oxidation potential and high electron transfer rates. Especially after the recent global COVID-19 pandemic, demand for POC devices is rising steadily [120]. AgNPs have the potential of reaching the required high standards of low LODs, high stability, and easy handling. These properties are further highlighted by the literature discussed in Table 2, which gives further examples of recent promising biosensing strategies including AgNPs. Furthermore, considering the practical necessity of scaling up nanoparticle synthesis, AgNPs currently are the most produced metallic nanomaterial [119]. Synthesis of specialized nanoparticles for novel biosensors in large batches can therefore be achieved quickly and inexpensively, simplifying the transfer of POC devices developed in laboratories into commercially available devices in contrast to the challenges of scale-up observed for many other nanomaterials. Considering the well-established detection principles based on AgNPs and AgNP composite materials for biosensors, their integration into existing or to-be-developed POC devices should be a likely and is a foreseeable next step that needs attention in the scientific community. AgNPs certainly have the potential of becoming a key material in this growing market in the near future.

**Table 2:** Select recent (2018–2023) biosensors incorporating silver nanoparticles. Papers were selected to represent an overview over the various kinds of use of silver nanoparticles in biosensors as presented in this review as well as represent some of the most interesting or promising examples of biosensing with AgNPs

Sensor type	Detection technique	Target analyte	Role of integrated AgNP	Linear range	LOD	Notes	References
Optical biosensor	Fluorescence spectroscopy	Prostate-specific antigen	Amplificator of fluorophore	-	15.66 pg mL^−1^	Hydrogen peroxide etching of AgNPs to Ag^+^ turns on the fluorophore	[121]
		Metformin hydrochlorine	Inner filter effect with carbon dots for fluorescence quenching	2–100 µg L^−1^	1.76 µg L^−1^	Carbon dot/silver nanoparticle nanocomposites	[122]
		HIV-1 p24 antigen	Fluorescent label	10–1000 pg mL^−1^	8.2 pg mL^−1^	Fluorescent silver nanoparticles modified with streptavidin couple to biotinylated detector antibody	[123]
	Fluorescence + UV/Vis spectroscopy	Parathion methyl	Fluorescence quencher Colorimetric label	0.1–6 µg L^−1^	0.017 µg L^−1^		[124]
	UV/Vis-Spectroscopy	Ni^2+^ ions	Colorimetric label (SPR)	2.15 µM	3–7 µM		[125]
		Cholesterol	Colorimetric label		40 µM	Chemosensor for hydrogen peroxide and biosensor (with cholesterol oxidase) for cholesterol detection	[126]
		Cu^2+^ ions	Part of sensing system consisting of AuNPs, AgNPs, and thymine	0.03–0.5 ppm	0.03 ppm	Smartphone readout possible (LOD: 0.09 ppm)	[127]
	Raman-Spectroscopy (SERS)	*Salmonella*	SERS substrate	10–10^5^ CFU mL^−1^	6 CFU mL^−1^	Combination of hybridization chain reaction with SERS	[128]
		Dopamine	SERS substrate		8.3 nM (in presence of ascorbic acid and uric acid)		[129]
		Urea	SERS substrate	8.25–825 nM	4.92 nM		[130]
Optical + electrochemical biosensor	UV/Vis spectroscopy/Colorimetry Voltammetry (CV)	Hydrogen peroxide (in Milk)	SPR label (aggregation) Electrochemical label	0.5–5000 µM	3.84 µM (colorimetric) 33.52 nM (electrochemical)	Polyphenol extract/Silver nanoparticles (PPE/AgNPs) used in both colorimetric sensor (On cotton fabric + smartphone readout and electrochemical sensor)	[131]
Electrochemical biosensor	Voltammetry (CV)	*Salmonella*	AgNP-rGO nanocomposite for electrode modification	10–10^5^ CFU mL^−1^	22 CFU mL^−1^		[132]
		Urea	Electrocatalytic properties of Ag with high surface area of the NP	1–8 mM	0.14 mM	Deposition of AgNPs on commercially available glucose test strips as cheap electrodes	[133]
	Amperometry	Glyphosphate	Electrode material; signal enhancement; immobilization support	0.05 – 0.5 µg mL^−1^ 0.5 – 22.0 µg mL^−1^	0.015 µg mL^−1^		[134]
		Hydrogen peroxide	Signal enhancement on electrode	1.0–500 µM	0.35 µM	Electrode modification with photo-crosslinked horseradish peroxidase-polyglutamic acid-AgNP nanocomposites	[135]

## Conclusion

Long overlooked, AgNPs find increasing interest in optical and electrochemical biosensors. In comparison to AuNPs, AgNPs have a stronger surface plasmon resonance rendering them superior in optical sensing based on SPR, FRET, MEF, and SERS. Moreover, the less noble silver is oxidized using less toxic and aggressive chemicals (chemical dissolution) or at a considerably lower potential (electrochemical dissolution) minimizing matrix interferences in electrochemical sensing. Its higher electrical conductivity is highly beneficial for electrode modification strategies. Green synthesis strategies of AgNPs are well documented by now, and processes available to provide long-term storage and stability are available. Thus, for certainty, AgNPs are ready for their application in the POC field. Advantages as well as limitations for all of the detection technique used are summarized in Table 1. Moreover, solutions for these limitations, either already done by researchers or possible future developments, are listed together with recent literature examples for each detection technique. It can be deduced that overall, electrochemical detection through AgNPs shows many advantages over optical methods. However, it also becomes obvious that most formats proposed use experimental procedures that are too complicated to adapt for POC testing and more innovation is direly needed to overcome multistep processes. Also, long incubation times and the need for elevated assay temperatures hinder AgNP’s use in self-testing. We therefore propose that significant efforts are needed to develop actual POC-ready formats to use the highly beneficial properties of AgNPs to their full extend. In contrast, so much nanoparticle-related research is undertaken toward the invention and identification of new compositions or combinations of materials to develop ultrasensitive sensors rather than facilitating the experimental procedures and finding a real application for the existing materials and developing this further. Considering the exciting examples described here, we conclude though that AgNPs are more than ready and have a great potential as signal generator and signal amplification system and may serve as game changers in developing POCTs to be sensitive and quantitative enough for many of the challenging biomarkers.

## Data Availability

Data sharing not applicable to this article as no datasets were generated or analysed during the current study.

## References

[1] Beyene HD, Werkneh AA, Bezabh HK, Ambaye TG (2017). Synthesis paradigm and applications of silver nanoparticles (AgNPs), a review. Sustain Mater Technol.

[2] Loiseau A, Asila V, Boitel-Aullen G, Lam M, Salmain M, Boujday S (2019) Silver-based plasmonic nanoparticles for and their use in biosensing. Biosensors 9. 10.3390/bios9020078 10.3390/bios9020078 PMC6627098 31185689

[3] Marin S, Vlasceanu GM, Tiplea RE, Bucur IR, Lemnaru M, Marin MM, Grumezescu AM (2015). Applications and toxicity of silver nanoparticles: a recent review. Curr Top Med Chem.

[4] Flores-López LZ, Espinoza-Gómez H, Somanathan R (2019). Silver nanoparticles: electron transfer, reactive oxygen species, oxidative stress, beneficial and toxicological effects. Mini review. J Appl Toxicol JAT.

[5] Siddiqi KS, Husen A, Rao RAK (2018). A review on biosynthesis of silver nanoparticles and their biocidal properties. J Nanobiotechnol.

[6] Rafique M, Sadaf I, Rafique MS, Tahir MB (2017). A review on green synthesis of silver nanoparticles and their applications. Artif Cells Nanomed Biotechnol.

[7] Benn TM, Westerhoff P (2008). Nanoparticle silver released into water from commercially available sock fabrics. Environ Sci Technol.

[8] Kaur P, Luthra R (2016). Silver nanoparticles in dentistry: an emerging trend. SRM J Res Dent Sci.

[9] Xu L, Wang Y-Y, Huang J, Chen C-Y, Wang Z-X, Xie H (2020). Silver nanoparticles: synthesis, medical applications and biosafety. Theranostics.

[10] Durán N, Silveira CP, Durán M, Martinez DST (2015). Silver nanoparticle protein corona and toxicity: a mini-review. J Nanobiotechnol.

[11] Zhang X-F, Liu Z-G, Shen W, Gurunathan S (2016) Silver nanoparticles: synthesis, characterization, properties, applications, and therapeutic approaches. Int J Mol Sci 17. 10.3390/ijms17091534 10.3390/ijms17091534 PMC5037809 27649147

[12] Bhakta SA, Evans E, Benavidez TE, Garcia CD (2015). Protein adsorption onto nanomaterials for the development of biosensors and analytical devices: a review. Anal Chim Acta.

[13] Rawat M (2016) A review on green synthesis and characterization of silver nanoparticles and their applications: a green nanoworld. WJPPS 730–762. 10.20959/wjpps20167-7227

[14] Fahmy HM, Mosleh AM, Elghany AA, Shams-Eldin E, Abu Serea ES, Ali SA, Shalan AE (2019). Coated silver nanoparticles: synthesis, cytotoxicity, and optical properties. RSC Adv.

[15] Tarannum N, Divya D, Gautam YK (2019). Facile green synthesis and applications of silver nanoparticles: a state-of-the-art review. RSC Adv.

[16] Kaabipour S, Hemmati S (2021). A review on the green and sustainable synthesis of silver nanoparticles and one-dimensional silver nanostructures. Beilstein J Nanotechnol.

[17] Yang SM, Kim SRN, Youn WK, Kim CS, Kim DS, Yi KW, Hwang NM (2015). Generation of charged nanoparticles during thermal evaporation of silver at atmospheric pressure. J Nanosci Nanotechnol.

[18] Chen Y-H, Yeh C-S (2002). Laser ablation method: use of surfactants to form the dispersed Ag nanoparticles. Colloids Surf A.

[19] Khayati GR, Janghorban K (2012). The nanostructure evolution of Ag powder synthesized by high energy ball milling. Adv Powder Technol.

[20] Ashkarran AA (2010). A novel method for synthesis of colloidal silver nanoparticles by arc discharge in liquid. Curr Appl Phys.

[21] Tien D-C, Tseng K-H, Liao C-Y, Huang J-C, Tsung T-T (2008). Discovery of ionic silver in silver nanoparticle suspension fabricated by arc discharge method. J Alloy Compd.

[22] Lee SH, Jun B-H (2019) Silver nanoparticles: synthesis and application for nanomedicine. Int J Mol Sci 20. 10.3390/ijms20040865 10.3390/ijms20040865 PMC6412188 30781560

[23] Iravani S, Korbekandi H, Mirmohammadi SV, Zolfaghari B (2014). Synthesis of silver nanoparticles: chemical, physical and biological methods. Res Pharm Sci.

[24] Khaydarov RA, Khaydarov RR, Gapurova O, Estrin Y, Scheper T (2009). Electrochemical method for the synthesis of silver nanoparticles. J Nanopart Res.

[25] Gu Y, Li N, Gao M, Wang Z, Xiao D, Li Y, Jia H, He H (2015). Microwave-assisted synthesis of BSA-modified silver nanoparticles as a selective fluorescent probe for detection and cellular imaging of cadmium(II). Microchim Acta.

[26] Zhang J, Chen P, Sun C, Hu X (2004). Sonochemical synthesis of colloidal silver catalysts for reduction of complexing silver in DTR system. Appl Catal A.

[27] Li T, Moon J, Morrone AA, Mecholsky JJ, Talham DR, Adair JH (1999). Preparation of Ag/SiO 2 nanosize composites by a reverse micelle and sol−gel technique. Langmuir.

[28] Goulet PJG, Lennox RB (2010). New insights into Brust-Schiffrin metal nanoparticle synthesis. J Am Chem Soc.

[29] Brust M, Walker M, Bethell D, Schiffrin DJ, Whyman R (1994). Synthesis of thiol-derivatised gold nanoparticles in a two-phase Liquid-Liquid system. J Chem Soc Chem Commun.

[30] Shafey AME (2020). Green synthesis of metal and metal oxide nanoparticles from plant leaf extracts and their applications: a review. Green Process Synth.

[31] Iravani S (2011). Green synthesis of metal nanoparticles using plants. Green Chem.

[32] Du H, Li Z, Wang Y, Yang Q, Wu W (2020) Nanomaterial-based optical biosensors for the detection of foodborne bacteria. Food Rev Int 1–30. 10.1080/87559129.2020.1740733

[33] Xia N, Wang X, Zhou B, Wu Y, Mao W, Liu L (2016). Electrochemical detection of amyloid-β oligomers based on the signal amplification of a network of silver nanoparticles. ACS Appl Mater Interfaces.

[34] Luppa PB (2012). POCT - Patientennahe Labordiagnostik.

[35] Vashist SK (2017) Point-of-care diagnostics: recent advances and trends. Biosensors 7. 10.3390/bios7040062 10.3390/bios7040062 PMC5746785 29258285

[36] Guo X (2012). Surface plasmon resonance based biosensor technique: a review. J Biophotonics.

[37] Li M, Li R, Li CM, Wu N (2011). Electrochemical and optical biosensors based on nanomaterials and nanostructures: a review. Front Biosci (Schol Ed).

[38] Jeong Y, Kook Y-M, Lee K, Koh W-G (2018). Metal enhanced fluorescence (MEF) for biosensors: general approaches and a review of recent developments. Biosens Bioelectron.

[39] Ghosh D, Chattopadhyay N (2015). Gold and silver nanoparticles based superquenching of fluorescence: a review. J Lumin.

[40] Ravindran A, Chandran P, Khan SS (2013). Biofunctionalized silver nanoparticles: advances and prospects. Colloids and surfaces. B Biointerfaces.

[41] Yu L, Li N (2019). Noble metal nanoparticles-based colorimetric biosensor for visual quantification: a mini review. Chemosensors.

[42] Diamai S, Negi DPS (2019). Cysteine-stabilized silver nanoparticles as a colorimetric probe for the selective detection of cysteamine. Spectrochimica acta. Part A Mol Biomol Spectrosc.

[43] Thomas A, Sivasankaran U, Kumar KG (2018). Biothiols induced colour change of silver nanoparticles: a colorimetric sensing strategy. Spectrochimica acta. Part A Mol Biomol Spectrosc.

[44] Ma X, Miao P (2019). Silver nanoparticle@DNA tetrahedron-based colorimetric detection of HIV-related DNA with cascade strand displacement amplification. J Mater Chem B.

[45] Yousefi S, Saraji M (2019). Optical aptasensor based on silver nanoparticles for the colorimetric detection of adenosine. Spectrochimica acta. Part A Mol Biomol Spectrosc.

[46] Wang X, Hou T, Lin H, Lv W, Li H, Li F (2019). In situ template generation of silver nanoparticles as amplification tags for ultrasensitive surface plasmon resonance biosensing of microRNA. Biosens Bioelectron.

[47] Grady M, Pineau M, Pynes MK, Katz LB, Ginsberg B (2014). A clinical evaluation of routine blood sampling practices in patients with diabetes: impact on fingerstick blood volume and pain. J Diabetes Sci Technol.

[48] Shariati S, Khayatian G (2021). The colorimetric and microfluidic paper-based detection of cysteine and homocysteine using 1,5-diphenylcarbazide-capped silver nanoparticles. RSC Adv.

[49] Bélteky P, Rónavári A, Igaz N, Szerencsés B, Tóth IY, Pfeiffer I, Kiricsi M, Kónya Z (2019). Silver nanoparticles: aggregation behavior in biorelevant conditions and its impact on biological activity. Int J Nanomed.

[50] Choi C-K, Shaban SM, Moon B-S, Pyun D-G, Kim D-H (2021). Smartphone-assisted point-of-care colorimetric biosensor for the detection of urea via pH-mediated AgNPs growth. Analytica chimica acta.

[51] Zhao P, Li H-X, Li D-W, Hou Y-J, Mao L, Yang M, Wang Y (2019). A SERS nano-tag-based magnetic-separation strategy for highly sensitive immunoassay in unprocessed whole blood. Talanta.

[52] Pang Y, Wang C, Lu L, Wang C, Sun Z, Xiao R (2019). Dual-SERS biosensor for one-step detection of microRNAs in exosome and residual plasma of blood samples for diagnosing pancreatic cancer. Biosens Bioelectron.

[53] Chen J, Wu Y, Fu C, Cao H, Tan X, Shi W, Wu Z (2019). Ratiometric SERS biosensor for sensitive and reproducible detection of microRNA based on mismatched catalytic hairpin assembly. Biosens Bioelectron.

[54] Bu Y, Zhu G, Li S, Qi R, Bhave G, Zhang D, Han R, Sun D, Liu X, Hu Z, Liu X (2018). Silver-nanoparticle-embedded porous silicon disks enabled SERS signal amplification for selective glutathione detection. ACS Appl Nano Mater.

[55] Meng X, Wang H, Chen N, Ding P, Shi H, Zhai X, Su Y, He Y (2018). A graphene-silver nanoparticle-silicon sandwich sers chip for quantitative detection of molecules and capture, discrimination, and inactivation of bacteria. Anal Chem.

[56] Quarin S, Strobbia P (2021). Recent advances towards point-of-care applications of surface-enhanced raman scattering sensing. Front Chem.

[57] He Y, Yang X, Yuan R, Chai Y (2019). A novel ratiometric SERS biosensor with one Raman probe for ultrasensitive microRNA detection based on DNA hydrogel amplification. J Mater Chem B.

[58] Macdonald D, Smith E, Faulds K, Graham D (2020). DNA detection by SERS: hybridisation parameters and the potential for asymmetric PCR. Analyst.

[59] Ben-Jaber S, Peveler WJ, Quesada-Cabrera R, Cortés E, Sotelo-Vazquez C, Abdul-Karim N, Maier SA, Parkin IP (2016). Photo-induced enhanced Raman spectroscopy for universal ultra-trace detection of explosives, pollutants and biomolecules. Nat Commun.

[60] Man T, Lai W, Xiao M, Wang X, Chandrasekaran AR, Pei H, Li L (2020). A versatile biomolecular detection platform based on photo-induced enhanced Raman spectroscopy. Biosens Bioelectron.

[61] Jin F, Li H, Xu D (2019). Enzyme-free fluorescence microarray for determination of hepatitis B virus DNA based on silver nanoparticle aggregates-assisted signal amplification. Anal Chim Acta.

[62] Iqbal S, Shabaninezhad M, Hatshan M, Niraula PM, Abuhagr A, Alali H, Guda R, Kayani A (2018). Ion-implanted silver nanoparticles for metal-enhanced fluorescence. AIP Adv.

[63] Yun BJ, Kwon JE, Lee K, Koh W-G (2019). Highly sensitive metal-enhanced fluorescence biosensor prepared on electrospun fibers decorated with silica-coated silver nanoparticles. Sens Actuators B Chem.

[64] Li J, Xu J, Guo W, Zhong W, Li Q, Tan L, Shang L (2020). Ratiometric fluorescence sensors for heparin and heparinase based on enhanced excimer emission of perylene probe induced by cationic silver nanoparticles. Sensors Actuators B: Chem.

[65] Kim M, Kwon JE, Lee K, Koh W-G (2018). Signal-amplifying nanoparticle/hydrogel hybrid microarray biosensor for metal-enhanced fluorescence detection of organophosphorus compounds. Biofabrication.

[66] Afzalinia A, Mirzaee M (2020). Ultrasensitive fluorescent mirna biosensor based on a "sandwich" oligonucleotide hybridization and fluorescence resonance energy transfer process using an Ln(III)-MOF and Ag nanoparticles for early cancer diagnosis: application of central composite design. ACS Appl Mater Interfaces.

[67] Liu J, Wang L, Bao H (2019). A novel fluorescent probe for ascorbic acid based on seed-mediated growth of silver nanoparticles quenching of carbon dots fluorescence. Anal Bioanal Chem.

[68] Lu Q, Chen X, Liu D, Wu C, Liu M, Li H, Zhang Y, Yao S (2018). Synergistic electron transfer effect-based signal amplification strategy for the ultrasensitive detection of dopamine. Talanta.

[69] Jiang Y, Ma X, Shao X, Wang M, Jiang Y, Miao P (2019). Chameleon silver nanoclusters for ratiometric sensing of miRNA. Sensors Actuators B: Chem.

[70] Jiang Y, Tang Y, Miao P (2019). Polydopamine nanosphere@silver nanoclusters for fluorescence detection of multiplex tumor markers. Nanoscale.

[71] Wang C, Xing K, Zhang G, Yuan M, Xu S, Liu D, Chen W, Peng J, Hu S, Lai W-H (2019). Novel ELISA based on fluorescent quenching of DNA-stabilized silver nanoclusters for detecting E. coli O157:H7. Food Chem.

[72] Shen F, Cheng Y, Xie Y, Yu H, Yao W, Li H-W, Guo Y, Qian H (2019). DNA-silver nanocluster probe for norovirus RNA detection based on changes in secondary structure of nucleic acids. Anal Biochem.

[73] Del Bonis-O'Donnell JT, Thakrar A, Hirschberg JW, Vong D, Queenan BN, Fygenson DK, Pennathur S (2018). DNA-stabilized silver nanoclusters as specific, ratiometric fluorescent dopamine sensors. ACS Chem Neurosci.

[74] Yazdanparast S, Benvidi A, Banaei M, Nikukar H, Tezerjani MD, Azimzadeh M (2018). Dual-aptamer based electrochemical sandwich biosensor for MCF-7 human breast cancer cells using silver nanoparticle labels and a poly(glutamic acid)/MWNT nanocomposite. Mikrochim Acta.

[75] Pichaimuthu K (2018) Silver nanoparticles decorated on graphene oxide sheets for electrochemical detection of ascorbic acid(AA) in human urine sample. Int J Electrochem Sci 7859–7869. 10.20964/2018.08.16

[76] Miao X, Wang Y, Gu Z, Mao D, Ning L, Cao Y (2018). Cucurbit8uril-assisted peptide assembly for feasible electrochemical assay of histone acetyltransferase activity. Anal Bioanal Chem.

[77] Miao P, Jiang Y, Wang Y, Yin J, Tang Y (2018). An electrochemical approach capable of prostate specific antigen assay in human serum based on exonuclease-aided target recycling amplification. Sens Actuators B Chem.

[78] Abbaspour A, Norouz-Sarvestani F, Noori A, Soltani N (2015). Aptamer-conjugated silver nanoparticles for electrochemical dual-aptamer-based sandwich detection of staphylococcus aureus. Biosens Bioelectron.

[79] Yu C-X (2020) Electrochemical biosensors with silver nanoparticles as signal labels. Int J Electrochem Sci 3869–3890. 10.20964/2020.05.53

[80] Chen S, Yuan R, Chai Y, Hu F (2013). Electrochemical sensing of hydrogen peroxide using metal nanoparticles: a review. Microchim Acta.

[81] Gao J, Jia M, Xu Y, Zheng J, Shao N, Zhao M (2018). Prereduction-promoted enhanced growth of silver nanoparticles for ultrasensitive colorimetric detection of alkaline phosphatase and carbohydrate antigen 125. Talanta.

[82] de la Escosura-Muñiz A, Parolo C, Maran F, Mekoçi A (2011). Size-dependent direct electrochemical detection of gold nanoparticles: application in magnetoimmunoassays. Nanoscale.

[83] Song W, Li H, Liang H, Qiang W, Xu D (2014). Disposable electrochemical aptasensor array by using in situ DNA hybridization inducing silver nanoparticles aggregate for signal amplification. Anal Chem.

[84] Geagea R, Aubert P-H, Banet P, Sanson N (2015). Signal enhancement of electrochemical biosensors via direct electrochemical oxidation of silver nanoparticle labels coated with zwitterionic polymers. Chem Commun (Camb).

[85] Chai H, Miao P (2019). Bipedal DNA walker based electrochemical genosensing strategy. Anal Chem.

[86] Beck F, Horn C, Baeumner AJ (2022). Ag nanoparticles outperform Au nanoparticles for the use as label in electrochemical point-of-care sensors. Anal Bioanal Chem.

[87] Pollok NE, Rabin C, Walgama CT, Smith L, Richards I, Crooks RM (2020). Electrochemical detection of NT-proBNP using a metalloimmunoassay on a paper electrode platform. ACS Sensors.

[88] Walgama C, Nguyen MP, Boatner LM, Richards I, Crooks RM (2020). Hybrid paper and 3D-printed microfluidic device for electrochemical detection of Ag nanoparticle labels. Lab Chip.

[89] Ma W, Ma H, Yang Z-Y, Long Y-T (2018). Single Ag nanoparticle electro-oxidation: potential-dependent current traces and potential-independent electron transfer kinetic. J Phys Chem Lett.

[90] Beck F, Horn C, Baeumner AJ (2022). Dry-reagent microfluidic biosensor for simple detection of NT-proBNP via Ag nanoparticles. Analytica chimica acta.

[91] Song S, Hu X, Li H, Zhao J, Koh K, Chen H (2018). Guests involved CB[8] capped silver nanoparticles as a means of electrochemical signal enhancement for sensitive detection of Caspase-3. Sens Actuators B Chem.

[92] Chen X, Wang Y, Zhang J, Zhang Y (2019). DNA concatemer-silver nanoparticles as a signal probe for electrochemical prostate-specific antigen detection. Analyst.

[93] Cheng W, Ma J, Kong D, Zhang Z, Khan A, Yi C, Hu K, Yi Y, Li J (2021). One step electrochemical detection for matrix metalloproteinase 2 based on anodic stripping of silver nanoparticles mediated by host-guest interactions. Sensors Actuators B: Chem.

[94] Tang Y, Dai Y, Huang X, Li L, Han B, Cao Y, Zhao J (2019). Self-assembling peptide-based multifunctional nanofibers for electrochemical identification of breast cancer stem-like cells. Anal Chem.

[95] Asadzadeh-Firouzabadi A, Zare HR (2018). Preparation and application of AgNPs/SWCNTs nanohybrid as an electroactive label for sensitive detection of miRNA related to lung cancer. Sens Actuators B Chem.

[96] Meng F, Sun H, Huang Y, Tang Y, Chen Q, Miao P (2019). Peptide cleavage-based electrochemical biosensor coupling graphene oxide and silver nanoparticles. Anal Chim Acta.

[97] Chen P, Wang T, Zheng X, Tian D, Xia F, Zhou C (2018). An ultrasensitive electrochemical immunosensor based on C 60 -modified polyamidoamine dendrimers and Au NPs for co-catalytic silver deposition. New J Chem.

[98] Wang J, Liu X, Wang C, Liu D, Li F, Wang L, Liu S (2020). An integral recognition and signaling for electrochemical assay of protein kinase activity and inhibitor by reduced graphene oxide-polydopamine-silver nanoparticle-Ti4+ nanocomposite. Front Bioeng Biotechnol.

[99] Shamsipur M, Emami M, Farzin L, Saber R (2018). A sandwich-type electrochemical immunosensor based on in situ silver deposition for determination of serum level of HER2 in breast cancer patients. Biosens Bioelectron.

[100] Sun H, Kong J, Wang Q, Liu Q, Zhang X (2019). Dual signal amplification by eATRP and DNA-templated silver nanoparticles for ultrasensitive electrochemical detection of nucleic acids. ACS Appl Mater Interfaces.

[101] Sun H, Xu W, Liu B, Liu Q, Wang Q, Li L, Kong J, Zhang X (2019). Ultrasensitive detection of DNA via SI-eRAFT and in situ metalization dual-signal amplification. Anal Chem.

[102] Marques RC, Costa-Rama E, Viswanathan S, Nouws HP, Costa-García A, Delerue-Matos C, González-García MB (2018). Voltammetric immunosensor for the simultaneous analysis of the breast cancer biomarkers CA 15–3 and HER2-ECD. Sens Actuators B Chem.

[103] Khalifa Z, Zahran M, Zahran MA-H, Azzem MA (2020). Mucilage-capped silver nanoparticles for glucose electrochemical sensing and fuel cell applications. RSC Adv..

[104] Campbell FW, Compton RG (2010). The use of nanoparticles in electroanalysis: an updated review. Anal Bioanal Chem.

[105] Rosati G, Cunego A, Fracchetti F, Del Casale A, Scaramuzza M, de Toni A, Torriani S, Paccagnella A (2019). Inkjet Printed interdigitated biosensor for easy and rapid detection of bacteriophage contamination: a preliminary study for milk processing control applications. Chemosensors.

[106] Maduraiveeran G, Kundu M, Sasidharan M (2018). Electrochemical detection of hydrogen peroxide based on silver nanoparticles via amplified electron transfer process. J Mater Sci.

[107] El Badawy AM, Scheckel KG, Suidan M, Tolaymat T (2012). The impact of stabilization mechanism on the aggregation kinetics of silver nanoparticles. Sci Total Environ.

[108] Reidy B, Haase A, Luch A, Dawson KA, Lynch I (2013). Mechanisms of silver nanoparticle release, transformation and toxicity: a critical review of current knowledge and recommendations for future studies and applications. Materials (Basel Switzerland).

[109] Beer C, Foldbjerg R, Hayashi Y, Sutherland DS, Autrup H (2012). Toxicity of silver nanoparticles - nanoparticle or silver ion?. Toxicol Lett.

[110] Newton KM, Puppala HL, Kitchens CL, Colvin VL, Klaine SJ (2013). Silver nanoparticle toxicity to Daphnia magna is a function of dissolved silver concentration. Environ Toxicol Chem.

[111] Pakrashi S, Tan C, Wang W-X (2017). Bioaccumulation-based silver nanoparticle toxicity in Daphnia magna and maternal impacts. Environ Toxicol Chem.

[112] Ivask A, Elbadawy A, Kaweeteerawat C, Boren D, Fischer H, Ji Z, Chang CH, Liu R, Tolaymat T, Telesca D, Zink JI, Cohen Y, Holden PA, Godwin HA (2014). Toxicity mechanisms in Escherichia coli vary for silver nanoparticles and differ from ionic silver. ACS Nano.

[113] Titov EA, Sosedova LM, Novikov MA, Zvereva MV, Rukavishnikov VS, Lakhman OL (2022) The analysis of acute and subacute toxicity of silver selenide nanoparticles encapsulated in arabinogalactan polymer matrix. Polymers 14. 10.3390/polym14153200 10.3390/polym14153200 PMC9370907 35956714

[114] de Luna LAV, de Moraes ACM, Consonni SR, Pereira CD, Cadore S, Giorgio S, Alves OL (2016). Comparative in vitro toxicity of a graphene oxide-silver nanocomposite and the pristine counterparts toward macrophages. J Nanobiotechnol.

[115] McShan D, Ray PC, Yu H (2014). Molecular toxicity mechanism of nanosilver. J Food Drug Anal.

[116] Ahamed M, Alsalhi MS, Siddiqui MKJ (2010). Silver nanoparticle applications and human health. Clinica Chimica Acta.

[117] Hadrup N, Lam HR (2014). Oral toxicity of silver ions, silver nanoparticles and colloidal silver–a review. Regul Toxicol Pharmacol RTP.

[118] Hadrup N, Sharma AK, Loeschner K (2018). Toxicity of silver ions, metallic silver, and silver nanoparticle materials after in vivo dermal and mucosal surface exposure: a review. Regul Toxicol Pharmacol RTP.

[119] Janković NZ, Plata DL (2019). Engineered nanomaterials in the context of global element cycles. Environ Sci Nano.

[120] Karako K, Song P, Chen Y, Tang W (2022). Increasing demand for point-of-care testing and the potential to incorporate the Internet of medical things in an integrated health management system. Biosci Trends.

[121] Peng W, Qin Y, Li W, Chen M, Zhou D, Li H, Cui J, Chang J, Xie S, Gong X, Tang B (2020). Nonenzyme cascaded amplification biosensor based on effective aggregation luminescence caused by disintegration of silver nanoparticles. ACS sensors.

[122] Le T-H, Kim J-H, Park S-J (2022) A co-doped carbon dot/silver nanoparticle nanocomposite-based fluorescence sensor for metformin hydrochloride detection. nanomaterials (Basel, Switzerland) 12. 10.3390/nano12081297 10.3390/nano12081297 PMC9030081 35458005

[123] Kurdekar AD, Chunduri LAA, Chelli SM, Haleyurgirisetty MK, Bulagonda EP, Zheng J, Hewlett IK, Kamisetti V (2017). Fluorescent silver nanoparticle based highly sensitive immunoassay for early detection of HIV infection. RSC Adv.

[124] Li Y, Chen S, Lin D, Chen Z, Qiu P (2020). A dual-mode nanoprobe for the determination of parathion methyl based on graphene quantum dots modified silver nanoparticles. Anal Bioanal Chem.

[125] Rossi A, Zannotti M, Cuccioloni M, Minicucci M, Petetta L, Angeletti M, Giovannetti R (2021) Silver nanoparticle-based sensor for the selective detection of nickel ions. nanomaterials (Basel, Switzerland) 11. 10.3390/nano11071733 10.3390/nano11071733 PMC8308118 34209361

[126] Tran HV, Nguyen TV, Nguyen LT, Hoang HS, Huynh CD (2020). Silver nanoparticles as a bifunctional probe for label-free and reagentless colorimetric hydrogen peroxide chemosensor and cholesterol biosensor. J Sci: Adv Mater Devices.

[127] Thongkam T, Apilux A, Tusai T, Parnklang T, Kladsomboon S (2022) Thy-AuNP-AgNP Hybrid systems for colorimetric determination of copper (II) ions using UV-Vis spectroscopy and smartphone-based detection. Nanomaterials (Basel, Switzerland) 12. 10.3390/nano12091449 10.3390/nano12091449 PMC9105095 35564160

[128] Li A, Zuo P, Ye B-C (2021). An aptamer biosensor based dual signal amplification system for the detection of salmonella typhimurium. Anal Biochem.

[129] Figueiredo ML, Martin CS, Furini LN, Rubira RJ, Batagin-Neto A, Alessio P, Constantino CJ (2020). Surface-enhanced Raman scattering for dopamine in Ag colloid: adsorption mechanism and detection in the presence of interfering species. Appl Surf Sci.

[130] Liang A, Wang H, Yao D, Jiang Z (2019). A simple and sensitive SERS quantitative analysis method for urea using the dimethylglyoxime product as molecular probes in nanosilver sol substrate. Food Chem.

[131] Karakuş S, Baytemir G, Taşaltın N (2022) Digital colorimetric and non-enzymatic biosensor with nanoarchitectonics of Lepidium meyenii-silver nanoparticles and cotton fabric: real-time monitoring of milk freshness. Appl Phys A 128. 10.1007/s00339-022-05529-6

[132] Vu QK, Nguyen TH, Le A-T, Vu NP, Ngo XD, Nguyen TK, Nguyen TT, van Pham C, Nguyen TL, Le Dang TT, Tonezzer M, Tran QH (2022). Enhancing electron transfer and stability of screen-printed carbon electrodes modified with AgNP-reduced graphene oxide nanocomposite. J Electron Mater.

[133] Liu J, Siavash Moakhar R, Sudalaiyadum Perumal A, Roman HN, Mahshid S, Wachsmann-Hogiu S (2020). An AgNP-deposited commercial electrochemistry test strip as a platform for urea detection. Sci Rep.

[134] Butmee P, Tumcharern G, Songsiriritthigul C, Durand MJ, Thouand G, Kerr M, Kalcher K, Samphao A (2021). Enzymatic electrochemical biosensor for glyphosate detection based on acid phosphatase inhibition. Anal Bioanal Chem.

[135] Xu S, Huang X, Chen Y, Liu Y, Zhao W, Sun Z, Zhu Y, Liu X, Wong C-P (2019). Silver nanoparticle-enzyme composite films for hydrogen peroxide detection. ACS Appl Nano Mater.

